# Peripheral Nerve Blocks Are Associated With Decreased Early Medical Complications, Dislocations, and Opioid Consumption Following Total Hip Arthroplasty

**DOI:** 10.1016/j.artd.2024.101587

**Published:** 2024-12-24

**Authors:** Brian P. McCormick, Sean B. Sequeira, Mark D. Hasenauer, Robert P. McKinstry, Frank R. Ebert, Henry R. Boucher

**Affiliations:** Department of Orthopaedic Surgery, MedStar Union Memorial Hospital, Baltimore, MD, USA

**Keywords:** Peripheral nerve block, Pain, Total hip arthroplasty

## Abstract

**Background:**

Peripheral nerve blocks (PNBs) may be utilized for postoperative pain control following total hip arthroplasty (THA). The purpose of this study was to evaluate the association between PNBs and postoperative complication rates, healthcare utilization, and opioid consumption following elective THA.

**Methods:**

Opioid-naive patients who received PNBs on the same day as undergoing THA for degenerative etiologies were identified from a large national database and matched 1:5 to a control cohort using propensity scoring. Rates of medical complications, inpatient readmissions, and emergency department presentations occurring within 90 days of THA and surgery-related complications occurring within 1 year of THA were compared using odds ratios. Total cost and perioperative opioid consumption in morphine milligram equivalents (MMEs) per day were also evaluated and compared between groups.

**Results:**

Propensity score matching resulted in 4748 PNB patients matched to 23,740 control patients. THA patients who received PNBs had lower incidences of deep vein thrombosis (odds ratio [OR] 0.67, *P* = .004), urinary tract infection (OR 0.76, *P* < .001), and dislocation (OR 0.51, *P* < .001). PNBs were also associated with decreased perioperative opioid consumption (38.6 MME/day vs 55.3 MME/day, *P* < .001). Regarding healthcare utilization, there were no differences between cohorts in rates of inpatient readmission, emergency department presentation, or total cost.

**Conclusions:**

PNBs are associated with decreased risk of deep vein thrombosis, urinary tract infection, and dislocation and decreased perioperative opioid consumption following THA.

**Level of evidence:**

III, Retrospective review.

## Introduction

Pain control following elective total hip arthroplasty (THA) remains a concern for orthopaedic surgeons. Poorly controlled pain after arthroplasty procedures is associated with mobility deficits, sleep disturbances, and increased complication rates [1,2]. A wide range of regional anesthetic techniques may be used to selectively block peripheral nerves and improve pain control and recovery following THA. Providers may therefore offer patients peripheral nerve blocks (PNBs) such as femoral nerve blocks, pericapsular nerve group (PENG) blocks, or fascia iliaca blocks to aid in postoperative pain control.

There has recently been significant interest in the utility of PNBs to aid in perioperative pain control during THA [[3], [4], [5], [6]]. A variety of regional anesthesia techniques exist to accomplish this goal. Femoral nerve and fascia iliaca blocks allow for improved postoperative pain control following elective THA, although motor function may also be affected [7,8]. The PENG block is a newly developed regional anesthesia technique that involves an ultrasound-guided injection of local anesthetic into the fascial plane between the psoas muscle and the superior pubic ramus in order to block the branches of the femoral and obturator nerves that provide sensory innervation to the hip joint capsule [[3], [4], [5], [6]]. A recent Cochrane review of these regional anesthesia techniques found decreased risk of complications, earlier mobilization, and decreased opioid consumption among hip fracture patients receiving PNBs [9]. While prior studies have shown promising results regarding the use of PNBs for pain control among elective hip arthroplasty patients, these studies have not been adequately powered to evaluate complication rates and healthcare utilization associated with this intervention. The purpose of this study was to compare early postoperative complication rates, healthcare utilization, and perioperative opioid consumption among patients who received a PNB block on the same day as elective THA compared to patients who did not.

## Material and methods

This is a retrospective cohort study utilizing the commercially available M161Ortho database via PearlDiver (PearlDiver Inc., Colorado Springs, Colorado). This database contains deidentified records for 161 million patients in the United States in accordance with the Health Insurance Portability and Accountability Act. Patient records were queried using International Classification of Diseases (ICD) codes and Current Procedural Terminology (CPT) codes. This study was deemed exempt from our institution’s review board as all available data from the M161Ortho database was already deidentified in accordance with Health Insurance Portability and Accountability Act.

Patients who underwent primary THA from 2010 through the second quarter of 2021 with at least 1 year of postoperative follow-up were identified using CPT and ICD codes. Patients previously diagnosed with chronic pain syndromes (ICD-9-D-33821, ICD-9-D-33829, ICD-9-D-3384, ICD-10-D-G8921, and ICD-10-D-G894) and patients who had filled prescriptions for opioids earlier than 1 week before surgery were excluded. A study group of opioid-naive patients who received nerve blocks on the same day as their index THA procedures was identified using relevant codes (CPT-64447 and CPT-64450). The code CPT-64447 includes patients who received a femoral nerve block, while CPT-64450 indicates an obturator nerve block. These codes were used together to capture a variety of peripheral nerve block techniques that may have targeted branches of the femoral nerve, obturator nerve, or a combination of both. These codes therefore represent a cohort of patients who may have received PENG blocks, fascia iliaca blocks, or femoral nerve blocks, as these procedures would all be accurately coded by CPT-64447 or CPT-64450. We aimed to evaluate patients who received single-shot regional anesthesia, so patients with the CPT code for a continuous femoral nerve catheter (CPT-64448) were excluded. Patients in the study group were matched to a control group using a propensity scoring methodology based upon Elixhauser Comorbidity Index (ECI) and Charlson comorbidity index (CCI) scores. ECI and CCI scores were then collected and compared to ensure successful propensity matching and similarity between cohorts (Table 1). To maintain patient anonymity, the PearlDiver database does not report outcomes with fewer than 10 patients. Complications with low incidences such as myocardial infarction and cerebrovascular accident were therefore not able to be reported as outcome measures.

**Table 1:** Demographic and comorbidity data.

Demographic/comorbidity data	PNB (n = 4748)	Control (n = 23,740)	P
Female sex (%)	2513 (52.9%)	13,870 (58.1%)	**<.001**
ECI (standard deviation)	3.44 (2.74)	3.44 (2.74)	1
CCI (standard deviation)	1.32 (1.81)	1.30 (1.80)	.485

Bold indicates *P* value <.05.

A study group of 4748 patients who received PNBs on the same date as undergoing THA were matched to 23,740 control patients using propensity scoring. ECI and CCI scores were similar between cohorts, although there was a significantly higher proportion of females in the control cohort (Table 1). Ninety-day incidences of deep vein thrombosis (DVT), pulmonary embolism, urinary tract infection (UTI), acute kidney injury, blood loss anemia, and pneumonia were evaluated as medical complications. One-year incidences of surgery-related complications were evaluated including dislocation, periprosthetic joint infection, all-cause revision surgery, periprosthetic fracture, component loosening, and wound dehiscence. Ninety-day incidences of emergency department presentation, hospital readmission, and total cost were evaluated as healthcare utilization outcomes. Hospital readmission was defined as occurring between 2 and 90 days postoperatively to avoid capturing patients with planned postoperative admissions. Opioid use in the perioperative period was compared using mean morphine milligram equivalents (MMEs) prescribed from 7 days before surgery to 90 days after surgery. Odds ratios (ORs) and 95% confidence intervals were calculated for each variable using R (University of Auckland, New Zealand). A *P*-value less than 0.05 was considered statistically significant.

## Results

Regarding 90-day medical complications, PNB patients had significantly lower incidences of DVT (OR 0.67, *P* = .004) and UTI (OR 0.76, *P* < .001) (Table 2). Regarding surgery-related complications, PNB patients had significantly lower rates of dislocation (OR 0.51, *P* < .001) (Table 3). There were otherwise no statistically significant differences in medical or surgery-related complications. There were no significant differences in healthcare utilization with similar rates of emergency department presentation (10.0% vs 10.4%), hospital readmission (4.4% vs 4.9%), or total cost ($9761 vs $9376) between cohorts (Table 4). Regarding opioid consumption, patients who received PNBs had significantly lower MMEs prescribed in the perioperative period compared to controls (38.6 MME/day vs 55.3 MME/day, *P* < .001) (Table 5).

**Table 2:** Ninety-day medical complications.

Complication	PNB (n = 4748)		Control (n = 23,740)		Statistical analysis		
	n	%	n	%	OR	95% CI	*P*
DVT	60	1.26%	443	1.87%	**0.67**	**0.51-0.88**	**.004**
PE	26	0.55%	182	0.77%	0.71	0.47-1.08	.107
UTI	193	4.06%	1261	5.31%	**0.76**	**0.65-0.88**	**<.001**
AKI	96	2.02%	488	2.06%	0.98	0.79-1.23	.881
Blood loss anemia	56	1.18%	328	1.38%	0.85	0.64-1.13	.271
PNA	71	1.50%	400	1.68%	0.89	0.69-1.14	.350

AKI, acute kidney injury; CI, confidence interval; PE, pulmonary embolism.

Bold indicates *P* value <.05.

**Table 3:** One-year surgery-related complications.

Complication	PNB (n = 4748)		Control (n = 23,740)		Statistical analysis		
	n	%	n	%	OR	95% CI	P
Dislocation	28	0.59%	272	1.15%	**0.51**	**0.35-0.76**	**<.001**
PJI	67	1.41%	342	1.44%	0.98	0.75-1.27	.876
All-cause revision	80	1.68%	429	1.81%	0.93	0.73-1.18	.562
Periprosthetic fracture	49	1.03%	213	0.90%	1.15	0.84-1.57	.375
Loosening	16	0.34%	131	0.55%	0.61	0.36-1.03	.062
Wound dehiscence	37	0.78%	132	0.56%	1.4	0.97-2.03	.069

PJI, periprosthetic joint infection.

Bold indicates *P* value <.05.

**Table 4:** Healthcare utilization.

Outcome measure	PNB (n = 4748)		Control (n = 23,740)		Statistical analysis		
	N	%	N	%	OR	95% CI	*P*
ED presentation	473	10.00%	2278	10.42%	1.04	0.94-1.16	.435
Hospital readmission	209	4.40%	1167	4.92%	0.89	0.77-1.04	.132
Total cost	9761 ± 26,520		9376 ± 16,282		-	-	.188

CI, confidence interval; ED, emergency department.

**Table 5:** Perioperative morphine milligram equivalents (MMEs) prescribed.

Opioid consumption	PNB (n = 4748)	Control (n = 23,740)	Difference, MME (95% CI)	*P*
Mean opioid consumption (MME/day)	38.6 ± 34.0	55.3 ± 58.9	**16.7 (14.9-18.5)**	**<.001**

CI, confidence interval.

Bold indicates *P* value <.05.

## Discussion

This study revealed that the administration of a PNB at the time of THA is associated with decreased risk of early postoperative complications and decreased perioperative opioid consumption. Interestingly, the rate of dislocation was approximately halved in the PNB group. These differences in complication rates may be attributed to improved pain control and mobility following THA with decreased opioid usage among patients receiving PNBs. Surgeons may be guided by this study in discussing the potential benefits of offering PNBs to elective THA patients, although future research is warranted to delineate the advantages and disadvantages of various PNB techniques.

This is the first study to our knowledge with an adequate sample size to evaluate postoperative complication rates among patients receiving PNBs at the time of elective THA. Several randomized controlled trials have investigated the efficacy of PNBs for improving pain control following THA, although these studies were not adequately powered to assess complications with low incidences [5,6,[10], [11], [12], [13], [14]]. Novel findings of this study include the decreased rates of DVT, UTI, and dislocation among patients who received PNBs (Table 2, Table 3). We hypothesize that the decreased incidences of DVT and UTI may be attributed to improved pain control and early mobilization, although the methodology of this study is not appropriate to determine the causation of these differences in complication rates. It similarly stands to reason that the low dislocation rate among PNB patients in this study may be attributed to their decreased opioid requirements, as multiple studies have shown opioid usage to be a risk factor for dislocation [[15], [16], [17]]. Various peripheral nerve blocks may have differing effects of early dislocation rates, especially given that some blocks are more likely to affect motor function than others [4,7,8]. Future research with adequately powered studies is therefore warranted to determine the complication rates associated with each PNB technique.

We found no difference in healthcare utilization among PNB patients compared to controls (Table 4). The cost of performing a PNB seems to have been offset by the decreased complication rates associated with this intervention. The decreased opioid consumption noted among PNB patients may have also contributed to this finding, as opioid use has been associated with an increased cost of arthroplasty procedures [18]. Our findings are consistent with some prior studies evaluated healthcare utilization among arthroplasty patients receiving PNBs, which have shown no difference in inpatient readmission rates [19], although two prior database studies found decreased readmission rates among patients who received femoral nerve blocks following total knee arthroplasty [20,21]. Further research is warranted to better understand the effect of various regional anesthetic techniques on healthcare utilization following THA.

There are several limitations to the current study. CPT codes representing femoral and obturator nerve blocks were utilized to identify patients who received PNBs. These CPT codes are nonspecific and included patients therefore would have received a variety of different types of PNBs, and we are unable to stratify results based upon the exact types of blocks that were administered. Furthermore, we isolated patients who received single-shot peripheral nerve blocks rather than patients who received regional analgesia through a continuous nerve catheter. Future research is warranted to better understand the utility and ideal patient population for each of these techniques. As a retrospective database study, our findings are dependent upon accurate diagnosis and coding of procedures, although the incidence of inaccuracy is estimated to be less than 1% [22]. Despite propensity score matching, there were differences in baseline demographics, as the control group had a higher percentage of female patients. The higher percentage of female patients in the control group may specifically have contributed to the increased rate of postoperative dislocation noted in this cohort. Furthermore, we were unable to match cohorts based upon variables not accounted for in the ECI and CCI score calculations such as payor status, practice setting, and demographic variables. Our methodology assumes an even distribution of PNBs performed across various practice environments, although PNBs may be more commonly performed at high-volume centers. This association may have contributed to the decreased rate of dislocation noted among patients receiving PNBs. We are similarly unable to stratify our results based on surgical approach, use of spinal anesthetic, implant design, or medication administered in the peripheral nerve block due to limitations inherent to this database. An advantage of the current study is having a sample size large enough to assess for differences in complication rates with low incidences. As a national database study, patients included would have received treatments from a variety of clinicians and environments, making our results broadly applicable to clinical practice.

## Conclusions

PNBs are associated with decreased risk of early medical and surgery-related complications following THA with decreased rates of postoperative DVT, UTI, and dislocation. We hypothesize that these differences in complication rates are due to improved pain control and decreased opioid consumption among PNB patients, although this study design was only able to identify these associations and is not appropriate to determine causality. Future research is warranted to better understand which regional anesthetic techniques offer the most significant benefits while mitigating risk among patients undergoing elective THA.

## CRediT authorship contribution statement

**Brian P. McCormick:** Writing – review & editing, Writing – original draft, Methodology, Formal analysis, Data curation, Conceptualization. **Sean B. Sequeira:** Writing – review & editing, Methodology, Formal analysis, Data curation, Conceptualization. **Mark D. Hasenauer:** Writing – review & editing, Visualization, Supervision, Methodology, Conceptualization. **Robert P. McKinstry:** Writing – review & editing, Visualization, Supervision, Methodology, Data curation, Conceptualization. **Frank R. Ebert:** Writing – review & editing, Visualization, Supervision, Project administration, Methodology, Conceptualization. **Henry R. Boucher:** Writing – review & editing, Visualization, Supervision, Project administration, Methodology, Investigation, Data curation, Conceptualization.

## Conflicts of interest

The authors declare there are no conflicts of interest.

For full disclosure statements refer to https://doi.org/10.1016/j.artd.2024.101587.

## Appendix A Supplementary data

Conflict of Interest Statement for McCormick

Conflict of Interest Statement for Ebert

Conflict of Interest Statement for Boucher

Conflict of Interest Statement for Hasenauer

Conflict of Interest Statement for McKinstry

Conflict of Interest Statement for Sequeira
